# Mendelian randomization study of causal link from gut microbiota to colorectal cancer

**DOI:** 10.1186/s12885-022-10483-w

**Published:** 2022-12-30

**Authors:** Jing-Jing Ni, Xiao-Song Li, Hong Zhang, Qian Xu, Xin-Tong Wei, Gui-Juan Feng, Min Zhao, Zi-Jia Zhang, Lei Zhang, Gen-Hai Shen, Bin Li

**Affiliations:** 1 Department of General Surgery, Suzhou Ninth Hospital Affiliated to Soochow University, 2666 Lu-dang Rd., Wujiang District, Jiangsu 215200 Suzhou, China; 2 Jiangsu Key Laboratory of Preventive and Translational Medicine for Geriatric Diseases, Medical College of Soochow University, Suzhou, Jiangsu China; 3 Center for Genetic Epidemiology and Genomics, School of Public Health, Medical College of Soochow University, 199 Ren-ai Rd., Jiangsu 215123 Suzhou, China; 4 Department of Epidemiology and Health Statistics, School of Public Health, Medical College of Soochow University, Jiangsu, China; 5 Inner Mongolia Medical University, Hohhot, Inner Mongolia China; 6 Inner Mongolia Autonomous Region People’s Hospital, Hohhot, Inner Mongolia China

**Keywords:** Mendelian randomization, Gut microbiota, Colorectal cancer, Causal relationship, genus *Blautia*

## Abstract

Recent studies have shown the relevance of gut microbiota in the occurrence and development of colorectal cancer (CRC), but the causal relationship remains unclear in the human population. The present study aims to assess the causal relationship from the gut microbiota to CRC and to identify specific causal microbe taxa via genome-wide association study (GWAS) summary statistics based two-sample Mendelian randomization (MR) analyses. Microbiome GWAS (MGWAS) in the TwinsUK 1,126 twin pairs was used as discovery exposure sample, and MGWAS in 1,812 northern German participants was used as replication exposure sample. GWAS of CRC in 387,156 participants from the UK Biobank (UKB) was used as the outcome sample. Bacteria were grouped into taxa features at both family and genus levels. In the discovery sample, a total of 30 bacteria features including 15 families and 15 genera were analyzed. Five features, including 2 families (*Verrucomicrobiaceae* and *Enterobacteriaceae*) and 3 genera (*Akkermansia*, *Blautia*, and *Ruminococcus*), were nominally significant. In the replication sample, the genus *Blautia* (discovery beta=-0.01, *P* = 0.04) was successfully replicated (replication beta=-0.18, *P* = 0.01) with consistent effect direction. Our findings identified genus *Blautia* that was causally associated with CRC, thus offering novel insights into the microbiota-mediated CRC development mechanism.

**Supplementary Information:**

The online version contains supplementary material available at 10.1186/s12885-022-10483-w.

## Introduction

Colorectal cancer (CRC) ranks the second and third in all cancers causing death in women and men, respectively, accounting for 10% of cancer-related deaths worldwide. The incidence of CRC is projected to reach 2.5 million in developing countries by 2035 [1]. Symptoms in early stage of CRC, including rectal bleeding, anemia and abdominal pain, are common to many other disorders, making early diagnosis of CRC difficult [2]. On the other hand, early diagnosis of CRC is vital for prolonged survival. The 5-year survival rate treated during the early-stage ranges from 72 to 100% while that during treatment at late-stage is quite poor [3].

The mechanism developing CRC is a multi-factorial process including genetics, environment, and their interaction [4]. Recent epidemiological studies indicate that colonic microbiota might affect colonic health via diet [5]. CRC patients harbor different microbial compositions compared to healthy volunteers [6, 7]. The fecal microbiota-based classification model has an accuracy of 0.798–0.93 to predict CRC in different classifiers [8]. Additional studies demonstrate that fecal short-chain fatty acids (SCFAs), the product of microbial protective metabolites, may exert potential anti-tumorigenic and anti-inflammatory effects [9], as confirmed by the modulation of colonic regulatory T cells in mice [10]. All these extensive endeavors imply that CRC is, at least in part, caused by abnormal microbiota metabolism. Nonetheless, the causal relationship between them is largely unknown in humans.

Mendelian randomization (MR) is an efficient approach to investigate the causal relationship from an exposure to an outcome in the cross-sectional study while controlling uncertain confounding effects [11, 12]. Conceptually, it is similar to a randomized controlled trial (RCT) in that genetic variables, as instrumental variables (IVs), are randomly assorted at birth into a “case” or “control” group and are fixed throughout their life, according to Mendel’s second law. The MR analysis assesses the association between the instrumental variables and the outcome which implies a causal association from exposure to outcome. To ensure the robustness of causal inference, the MR design relies on three essential assumptions: (i) single nucleotide polymorphisms (SNPs) are closely related to exposure; (ii) SNPs should be independent of any observed and unobserved confounders of exposure-outcome association; (iii) SNP-outcome association is only mediated by exposure and not through any other pathway. One recent study identified propionate as a mediator through which gut microbiota cause an increased risk of type 2 diabetes, demonstrating the efficacy of microbiota-oriented causal inference by MR analysis [13].

The routine MR approach utilizes individual-level information at both exposure and outcome sides. Restricted by limited experiment expense, individual-level data are usually small in sample size, limiting statistical power for testing causal association. As an alternative, summary statistics based MR analysis (as known as two-ample MR analysis) is approximately equivalent to individual-level MR analysis [14, 15]. Two-sample MR analysis utilizes SNP-exposure and SNP-outcome associations from two independent GWAS analyses and combines them into a single causal inference. Owing to the rapidly increasing amount of genome-wide association studies (GWAS) for both microbiota and complex diseases including cancers, large-scale GWAS summary statistics are becoming readily available [16–18], making it possible to implement summary statistics based MR analysis with largely improved statistical power over conventional individual-level based MR analysis.

In the present study, aiming to investigate the causal relationship from microbiota to CRC and to identify specific causal bacteria taxa, we conducted GWAS summary data based two-sample MR analyses. Specifically, summary data from 2 gut microbiota GWAS served as exposure (discovery + replication) while the GWAS of CRC in the UK Biobank (UKB) served as the outcome.

## Materials and methods

### Data sources

**Fig. 1:**
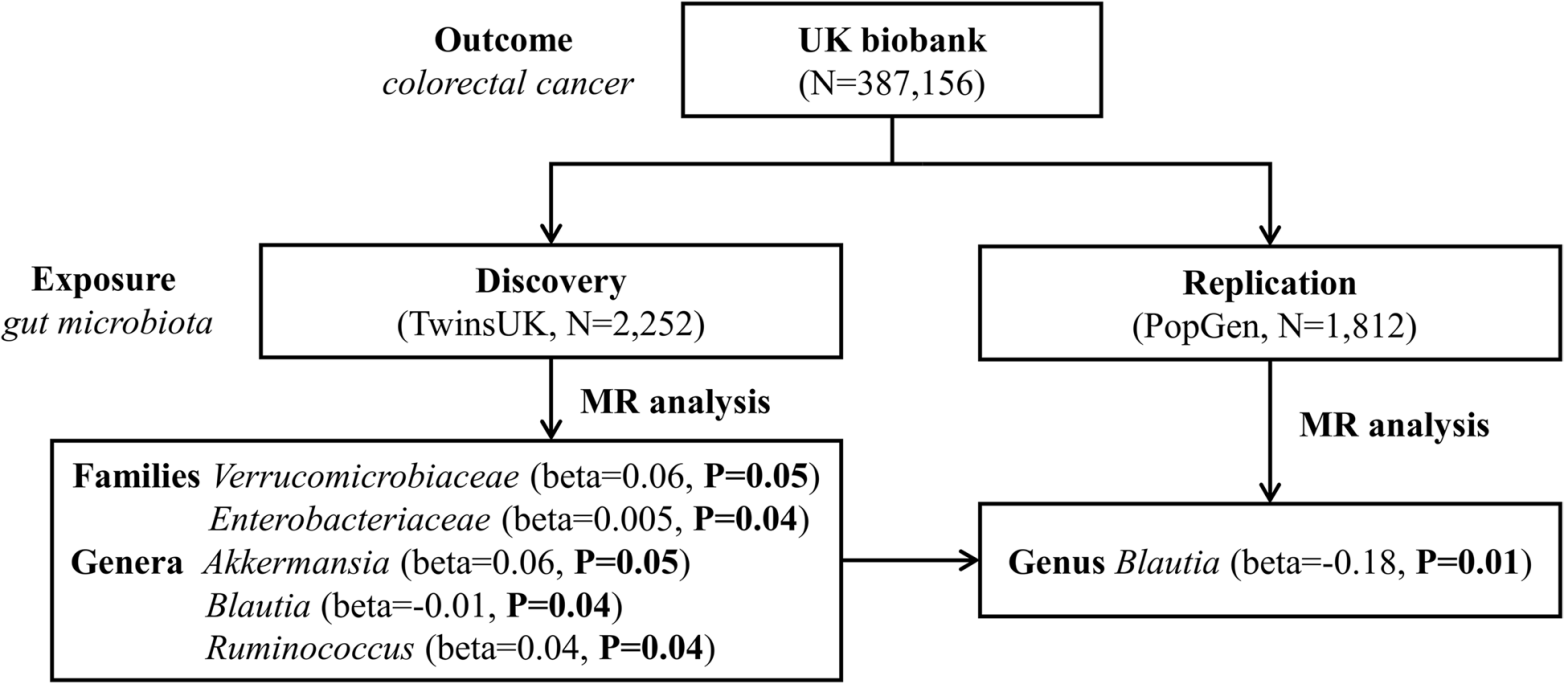
A flowchart of MR analysis in the discovery and replication. The MR analysis workflow and the main results were displayed in this figure

A flowchart briefly describes the whole procedure in Fig. 1. We conducted GWAS summary statistics based MR analysis. All studies were previously approved by respective institutional review boards (IRBs). No new IRB approval was required. Informed consent has been obtained from all participants and/or their legal guardians in their respective studies. The data were composed of gut microbiota and CRC GWAS summary statistics that were publicly available from previous studies or the corresponding authors.

The discovery gut microbiota sample was the TwinsUK study [16], a cohort of adult volunteer twins from the TwinsUK Registry in Britain. The data used in this study came from 1,126 twin pairs, as described elsewhere [16]. Briefly, 3,261 fecal samples were collected from all participants. The V4 hypervariable region of the 16 S rRNA gene from bulk DNA by PCR (primers 515 F and 806R) was amplified on all fecal samples, followed by purification and pooling. Microbiome 16 S rRNA was sequenced by the Illumina Miseq 2 × 250 bp platform, followed by classification via the Greengenes reference database and operational taxonomic units (OTUs) picking. Quality filtering and analysis of the 16 S rRNA gene sequence data were conducted with QIIME v1.7.0 and sequences with uncorrectable barcodes, ambiguous bases, or low-quality reads were removed, yielding a total of 302,554,236 sequences. The host genome was genotyped by Illumina HumanHap610 Quad Chip and was imputed into the 1000 Genomes project (phase 3) reference panel. Genetic association was examined between 945 bacteria taxa and 1.3 million imputed host SNPs. A total of 307 host SNPs were associated with 62 bacteria taxa (1 kingdom + 6 phyla + 9 classes + 10 orders + 16 families + 16 genera + 4 species) at a FDR < 0.2 and the *P*-values at these SNPs ranged from 4.94 × 10^− 9^ to 7.33 × 10^− 5^, as listed in Supplementary Table 1.

The replication gut microbiota sample was the PopGen study [17], a combined cohort of two separate samples PopGen and FoCus from northern Germany through the local Biobank PopGen. In brief, fecal samples were collected from 1,812 individuals of European ancestry in two independent but geographically matched cohorts. After bacteria DNA was extracted, the V1–V2 hypervariable region of the 16 S rRNA gene was sequenced on the MiSeq platform, using the 27 F-338R primer pair and dual MID indexing. Quality filtering was subsequently conducted using the fastx toolkit and UCHIME respectively, excluding sequences with more than 5% nucleotides (quality score < 30) and chimeras in sequences, followed by classification via RDP classifier based on the RDP14 reference database and species-level OTU creation by the UPARSE routine. Host genomes were genotyped by the Affymetrix Axiom array, custom Illumina Immunochip array, or Immumina Omni Express Exome array. The imputation was implemented by IMPUTE2 with the 1000 Genomes project (phase I) reference panel after excluding variants with a minor allele frequency (MAF) < 0.05. Genetic association of 64 bacteria taxa and 42 OTUs with host genotypes was examined with a generalized linear model, and the two sample were meta-analyzed. A total of 53 significant SNPs involving 40 loci and 36 bacterial traits (1 kingdom + 4 phyla + 7 classes + 8 orders + 8 families + 4 genera + 4 species) were identified at the genome-wide significance level (*P* < 5 × 10^− 8^) (Supplementary Table 2). Up to 13 bacteria taxa (3 phyla + 2 classes + 3 orders + 3 families + 2 genera) overlapped between these two gut microbiota cohorts, whereas the non-overlapping results could be attributable to limited statistical power and different methodologies by each cohort, among others. The features across the two studies were matched by looking for taxonomic names. Specifically, both studies aligned 16 S rRNA sequence data based on the pairwise alignment sequence dissimilarity metric and 97% similarity cutoff. OTUs representing taxonomical classification was then picked against known reference databases. Both databases hold sequence data of most of the known bacteria species and are not expected to have a major difference between them.

As outcome trait, the GWAS summary statistics for CRC in 387,156 UKB participants (4,562 cases and 382,756 controls) were utilized [18]. In brief, UKB is a prospective and population-based study among over 500,000 participants across the United Kingdom. CRC was diagnosed according to the International Classification of Disease diagnosis code 9 (ICD9). After imputation into the Haplotype Reference Consortium (HRC) reference panel, approximately 28 million genetic markers were available with minor allele counts (MACs) ≥ 20 and imputation info score ≥ 0.3. GWAS was performed in 387,156 qualified participants with Scalable and Accurate Implementation of GEneralized mixed model (SAIGE) for controlling unbalanced case-control ratio. The GWAS summary statistics were downloaded from the study’s website (https://www.leelabsg.org/resources).

### Patient and public involvement

This is a two-sample MR analysis based on GWAS summary data. The recruitments of all participants from gut microbiota GWAS in the TwinsUK study and the PopGen study, CRC GWAS in the UKB were implemented by their respective study. No additional recruitment was conducted. Patients were not involved in the recruitment, design and conduct of this study.

### Instrumental variable selection

Both discovery and replication exposure samples adopted same criteria for IV selection. Specifically, bacteria taxa were analyzed at both family and genus levels. A feature was defined as a distinct family or genus. As a quality control procedure, palindromic SNPs whose strand may be ambiguous were removed. The remaining SNPs were assigned to each feature based on their association significance for that feature. One feature may contain multiple bacterial taxa and thereby multiple association signals for one SNP. In this case, the signal with the strongest P-value was selected for the SNP. In accordance with Sanna et al. [13], SNP association threshold was set to be 1.0 × 10^− 5^. To account for linkage disequilibrium (LD) pattern, SNPs within each feature were clumped with PLINK (v1.9) [19] to retain independent SNPs only. The LD threshold was set to be r^2^ < 0.1 and the clumping window was set to be 500 kb. LD was estimated based on the 1000 genomes project sequencing data (phase 3).

The most severe confounding effect is the horizontal pleiotropy, which may violate the second assumption of MR design and confound the true causality, that is, the selected IVs are associated not only with microbiome taxa but also with other confounders such as BMI and age. To examine horizontal pleiotropy, the MR-PRESSO Global test and Outlier test [20] were applied. The MR-PRESSO Outlier test calculates for each SNP a *P*-value for its pleiotropy significance and the MR-PRESSO Global test calculates a *P*-value for overall horizontal pleiotropy. Evidence of pleiotropy significance was declared at a Bonferroni corrected *P*-value. All significant SNPs were removed. A MR-PRESSO Global test was finally applied to ensure no overall pleiotropic effect. The list of SNPs after removal of pleiotropic ones was used for subsequent MR analyses.

### MR analysis

We performed a two-sample MR analysis to integrate the information from both host-CRC and microbiome-host GWAS analyses, separately, and examine the causal from microbiome feature to the CRC outcome. Specifically, we tested association of the identified IVs within each microbiome feature with CRC. Four popular MR methods, including the inverse-variance weighted (IVW) test [21], the MR-Egger regression [22], the weighted median estimator [23], and the MR-PRESSO [20], were used for the MR analysis. The IVW method is reported to be slightly more powerful than the others under certain conditions [23]. Therefore, the results were mainly based on the IVW method while the other 3 methods served as its complements. For features containing only one IV for which the IVW test was not applicable, the Wald ratio test was used to estimate causal effect [24]. The potential heterogeneity was examined by the IVW test and the MR-Egger regression. Meanwhile, the leave-one-out sensitivity analysis was performed to examine if the causal signal was driven by one single SNP.

Significant features identified in the discovery TwinsUK study were subjected to be replicated in the replication PopGen sample with the same analysis procedure. All the above analyses including sensitivity analysis and MR analyses were performed with the R packages TwoSampleMR (https://github.com/MRCIEU/TwoSampleMR) [25] and MRPRESSO (https://github.com/rondolab/MR-PRESSO) [20].

## Results

In the discovery TwinsUK sample, after removing palindromic SNPs, there are a total of 245 SNPs associated with gut microbiota at the suggestive significance threshold p < 1.0 × 10^− 5^. After clumping, there are 171 and 81 SNPs left for families and genera, categorized into 15 families and 15 genera, respectively (Supplemental Table 3). The family with the largest number of SNPs is *Lachnospiraceae* (54 SNPs), followed by *Ruminococcaceae* (50 SNPs) and *Bacteroidaceae* (37 SNPs). There are 5 families, *Barnesiellaceae*, *Bifidobacteriaceae*, *Enterobacteriaceae*, *Streptococcaceae*, and *Veillonellaceae*, each containing only one SNP. At the genus level, the bacterium with the largest number of SNPs is *Bacteroides* (37 SNPs), followed by *Faecalibacterium* (9 SNPs) and *Coprococcus* (6 SNPs). There are 5 genera each containing only one SNP, *Anaerostipes*, *Bifidobacterium*, *Dorea*, *Streptococcus* and *Veillonella*. Of note, the genus is a child taxon of family, therefore the sets of SNPs contained in both features may heavily overlap. For example, the genus *Faecalibacterium* (9 SNPs) belongs to the family *Ruminococcaceae* (50 SNPs) and all the 9 SNPs overlap between them.

Sensitivity analysis was evaluated at all the included families and genera containing multiple IVs. There is no evidence of outlier or horizontal pleiotropy (both MR-PRESSO Global test p > 0.05/15 = 3.3 × 10^− 3^ and MR-Egger regression *p* > 0.05).

### MR analysis

In the discovery sample, the IVW MR analysis identifies two families *Verrucomicrobiaceae* (2 IVs, beta = 0.06, *P* = 0.05) and *Enterobacteriaceae* (1 IV, beta = 0.005, *P* = 0.04) that are causally associated with CRC risk. At the genus level, 3 bacteria taxa are causally associated at the nominal level, including *Akkermansia* (2 IVs, beta = 0.06, *P* = 0.05), *Blautia* (4 IVs, beta=-0.01, *P* = 0.04) and *Ruminococcus* (1 IV, beta = 0.01, *P* = 0.04). Most of these significant results are validated by the other 3 alternative MR tests, demonstrating the robustness across tests (Supplemental Table 4).

In total, 5 features (2 families + 3 genera) are causally associated with CRC in the discovery sample. Among them, the genus *Akkermansia* is within the family *Verrucomicrobiaceae*. Because no other genus within this family is included, both features contain exactly the same set of IVs and consequently result in the exact same *P*-values.

These 5 features are subjected to be replicated in the PopGen replication sample. In the replication sample, 53 SNPs were identified as IVs, 11 of which map to 2 of the above 5 features, while no SNP maps to the remaining 3 features. The 2 mapped features include family *Enterobacteriaceae* and genus *Blautia*. The same MR analysis successfully replicates genus *Blautia* (2 IVs, beta=-0.18, *P* = 0.01), while the family *Enterobacteriaceae* is not significant (9 IVs, beta=-0.005, *P* = 0.72). Of note, the effect direction of *Blautia* is consistent with that of the discovery sample (Table 1).

**Table 1:** Causal estimations of the gut microbiome on CRC in the discovery and replication cohorts

Stage	MR Test	Genus *Blautia*		
		No. SNP	b_xy_	*P*-value
Discovery				
	IVW	4	-0.01	**0.04**
	MR-Egger		-0.02	0.24
	Weighted Median		-0.01	0.11
	MR-PRESSO		-0.01	**0.04**
Replication				
	IVW	2	-0.18	**0.01**

No. SNP is the number of SNPs being used as IVs. b_xy_ is the estimated effect coefficient. Significant *P*-values were marked in bold. *IVW* inverse-variance weighted

**Fig. 2:**
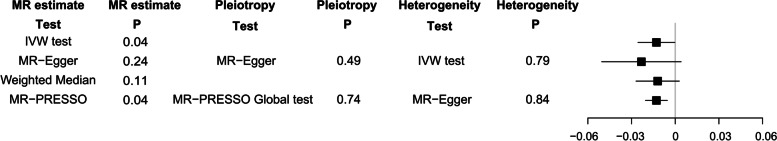
Forest plots of the 4 MR tests at the genus *Blautia*. Effect size, pleiotropy, and heterogeneity significance were displayed for each test, if applicable

**Fig. 3:**
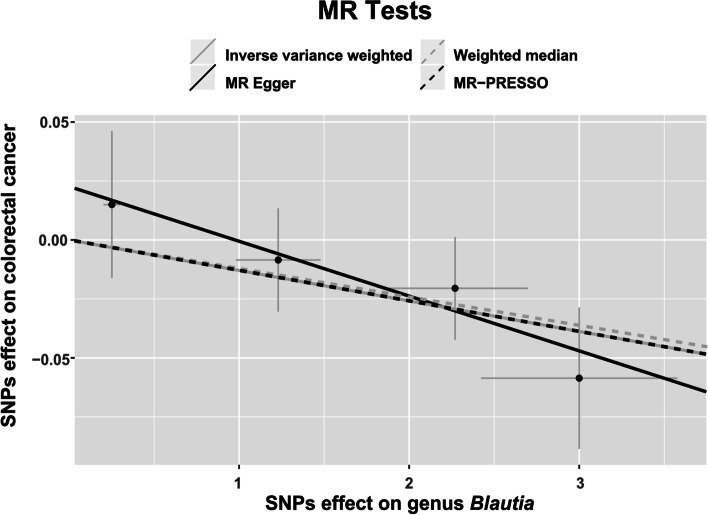
Scatter plot of the 4 MR tests at the genus *Blautia*. SNP effects were plotted into lines for the IVW test (grep solid line), MR-Egger regression (black solid line), weighted median estimator (grep dashed line), and MR-PRESSO (black dashed line). The slope of the line corresponded to the causal estimation

Neither the IVW test nor the MR-Egger test shows evidence of heterogeneity at the identified genus *Blautia* (P_IVW_=0.79; P_MR−Egger_=0.84). Furthermore, no evidence of horizontal pleiotropy is observed by either the MR-PRESSO test or the MR-Egger regression (P_MR−PRESSO Global_=0.74; P_MR−Egger_=0.49). Forest plots across various tests are displayed in Fig. 2 and the scatter plots are displayed in Fig. 3.

Furthermore, we have taken into account several potential confounders, inclduing diet (coffee intake, processed meat intake, bread intake, variation in diet and alcohol intake frequency) and obesity-related traits (obesity, BMI, weight, waist circumference, whole body fat mass, trunk fat mass, arm fat mass (left), arm fat mass (right), leg fat mass (left) and leg fat mass (right)) into consideration. We examined their associations with the selected IVs during the discovery and replication stages through the GeneATLAS website (http://geneatlas.roslin.ed.ac.uk/phewas). After multiple-testing correction (*P* < 0.05/(6 × 15) = 5.56 × 10^− 4^), the results showed that none of the associations is significant, as listed in the Supplementary Table 5.

**Fig. 4:**
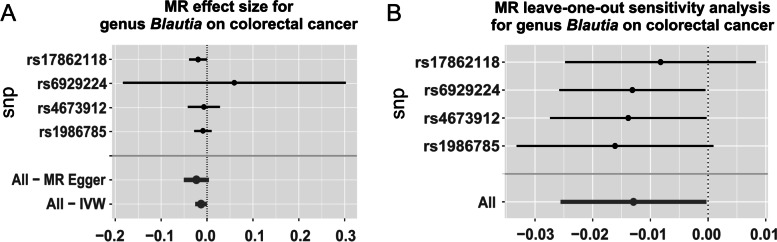
Forest plot of causal effects (**A**) and MR leave-one-out sensitivity analysis (**B**) for genus *Blautia* on colorectal cancer. **A** The causal effect of genus *Blautia* on colorectal cancer was estimated using each SNP singly using the Wald ratio, and using all SNPs using the MR Egger and IVW methods. **B** Leave-one-out sensitivity analysis represents the MR analysis excluding the particular SNP using the IVW test

In sum, genus *Blautia* is causally associated with CRC risk in the discovery sample (beta=-0.01, *P* = 0.04), and is successfully replicated in the replication sample (beta=-0.18, *P* = 0.01). The consistent effect direction strengths the confidence towards true association. A total of 6 SNPs are included as IVs in the discovery or replication sample and the detailed information of these SNPs were listed in Table 2. None of them is extremely significant for association with CRC and the leave-one-out sensitivity analysis demonstrates no single SNP driving the causal association signal, as displayed in Fig. 4A and B, respectively.

**Table 2:** SNPs detailed information of genus *Blautia* and colorectal cancer in the discovery and replication cohorts

Stage	SNP	Chr	Position	Locus	A1	A0	Closest gene	Exposure (genus *Blautia*)			Outcome (colorectal cancer)		
								Beta	SE	*P*-value	Beta	SE	*P*-value
Discovery													
	rs17862118	7	89,820,804	7q21.13	C	T	*STEAP2*	-3.00	0.578	2.40 × 10^− 7^	0.059	0.030	0.05
	rs1986785	13	30,029,722	13q12.3	C	A	*MTUS2*	-2.27	0.428	1.30 × 10^− 7^	0.021	0.022	0.35
	rs4673912	2	201,168,993	2q33.1	T	G	*SPATS2L*	1.23	0.249	8.34 × 10^− 7^	-0.009	0.022	0.70
	rs6929224	6	37,746,848	6p21.2	C	T	*ZFAND3*	2.52	0.050	4.28 × 10^− 7^	0.015	0.031	0.63
Replication													
	rs4669413	2	9,805,923	2p25.1	T	C	*YWHAQ*	-0.18	0.032	1.20 × 10^− 8^	0.040	0.022	0.07
	rs79387448	2	103,215,410	2q12.1	C	T	*SLC9A2*	-0.31	0.048	7.68 × 10^− 11^	0.048	0.029	0.10

Chr is Chromosome of SNP. Physical position is based on the human genome GRCH37 assembly. A1 is the effect allele and A0 is the other allele. Beta is the estimate coefficient of the effect allele. SE is the standard error of estimate coefficient. Closest gene is the closest gene to which the SNP mapped

## Discussion

In this study, we conducted a MR analysis to evaluate the causal relationship from gut microbiome to CRC. Using summary statistics from 2 microbiome GWAS and one CRC GWAS, we identified and replicated genus *Blautia* that was causally associated with CRC. The negative effect direction implied a protective regulation pattern.

The intestinal microbiota is an intricate and dynamic collection of ecological microbial communities that are colonized in the human gut, even called a “forgotten organ”. These bacteria play a crucial role in the homeostasis of the digestive system and the health of the host in multiple metabolic, immunological and protective functions [26]. The phylogenetic composition and function of intestinal bacteria are stable with age, while the diversity increases during growth. The large intestine comprises the densest and metabolism-active microorganism in healthy individuals, which are predominated by anaerobic microbiota, four phyla *Firmicutes* and *Bacteroidetes*, *Actinobacteria*, *Proteobacteria*, and *Verrucomicrobia* [27].

The genus *Blautia* identified in this study is a member of the family *Lachnospiraceae*, order *Clostridiales*, class *Clostridia* and phylum *Firmicutes*. It is characterized as gram-positive, non-motile and acetogenic strict anaerobe that mostly inhabits the intestinal tract in mammals [28]. Acetate, as one of the most abundant SCFAs, reaches relatively high concentration in peripheral blood [29]. It is accepted that SCFAs, produced by intestinal microbiota, inhibit the carcinogenesis of human colorectal cells [30]. Recent studies have shown that the abundance of *Blautia* gets decreased in CRC patients [31, 32]. Meanwhile, entire gut microbiome gets disrupted. For instance, *Firmicutes* is reduced in CRC group while *Bacteroidetes*, *Fusobacteria*, and *Proteobacteria* are enriched [33]. Furthermore, *Blautia obeum* might regulate dietary biotransformation of heterocyclic amines (HCA), so that HCA-induced CRC risk is decreased in a population-based case-control study [34]. As chronic inflammation and adenoma are risk factors for CRC [35, 36], genus *Blautia* shows the suppression of inflammation in the observational study [37] and adenoma reduction in CRC animal models [38, 39].

In this study, a total of 6 SNPs associated with genus *Blautia* are included in the discovery (containing rs17862118, rs1986785, rs4673912 and rs6929224) and replication (containing rs4669413 and rs79387448), which are located at different loci and genes. For instance, rs17862118, at the locus 7q21.13, is located within *STEAP2*, while *STEAP2* acts as a shuttle between the Golgi complex and the plasma membrane in the endocytic and exocytic pathways. STEAP2 is overexpressed in cancerous tissues such as prostate, bladder, colon and pancreas, but absent in vital organs [40] and may also affect uptake of iron and copper by proximal duodenal enterocytes [41]. rs6929224 is located at locus 6p21.2, and its closest gene is *ZFAND3*, a member of the ZFAND family of proteins containing the AN1 type ZF domain. ZF proteins ensure a variety of cellular functions in health and disease, such as DNA recognition, RNA packaging, and transcriptional regulation, and are implicated in many stages of cancer development [42]. The genetic regions and genes where these SNPs are located may contribute to partially explaining the potential mechanisms of how the genus *Blautia* affects the progression of colon carcinosis. The IV rs79387448 is located in the *SLC9A2* gene. An animal study [43] showed that *SLC9A2* expression is activated when colonic cells emerge from the stem cell niche which could affect enterocyte differentiation and electrolyte transport. Drew et al. [44] identified genetic markers, including *SLC9A2*, to distinguish between normal, adenomatous polyps and carcinomas, and real-time PCR, in-situ hybridization, and immunohistochemistry revealed aberrant epithelial expression of *SLC9A2* prior to carcinogenesis.

The MR method is an efficient approach for accessing the causal relationship from exposure to outcome while being robust to confounding effects. The MR performed in this study has the following advantages. First, it is a novel attempt to infer the causal relationship from gut microbiome to CRC, which provides a new approach to screen candidate gut microbiota for subsequent functional studies. Second, it is based on large-scale GWAS summary statistics that are publicly available, thus offers an efficient option to mine reliable genetic information without additional experimental costs.

Apparently, there are still several limitations in this study. Firstly, several typical CRC-associated taxa [45], such as *Fusobacterium nucleatum* and *Parvimonas*, are not identified in our study. The reason why is that these CRC-associated taxa are rare in general population and might not appear in the two gut microbiota GWAS of healthy participants. The SNPs associated with bacteria genera were also acquired independently from the CRC status. Therefore, their association with host genome is unlikely to be studied in the present study. Additionally, the mismatch of bacterial features across studies did not necessarily reflect the taxonomical heterogeneity, but a simple statistical matter. The SNPs\bacteria features used in the present study were those significantly associated ones instead of all the tested features from both microbiome GWAS studies. Because neither original study had perfect statistical power, each of them could only discovered a small fraction of all associated bacteria features. Secondly, gut microbiota GWAS is still in its infancy in terms of sample size, which provides insufficient information at the species or strain level. Furthermore, the loci identified so far are still extremely limited compared with the CRC GWAS area, which restricts the capacity to conduct a bidirectional MR analysis to infer a reverse causal relationship. We also noticed that despite being significant in both discovery and replication samples, the identified bacteria may still represent false positive signals, and further functional investigation is warranted to validate, which is out of the scope of the present study.

In conclusion, by conducting a two-sample MR analysis using publicly available GWAS summary data, we evaluated the causal link from gut microbiome to CRC as well as identified potentially causal bacteria taxa for colorectal carcinogenesis. This study may help to screen fecal microbial-based metabolites and markers for CRC early detection as non-invasive diagnostic or therapeutic targets, such as modulation of the gut microbiome and the transplantation of fecal microbiota.

## Supplementary Information

**Additional file 1.**

## Data Availability

No additional data. Data described in the manuscript, code book, and analytic code will be made available upon request pending application and approval from the principal investigator of PopGen and FoCus Study, Professor Andre Franke, and CRC GWAS of UK Biobank, Professor Seunggeun Lee, respectively. The data were composed of gut microbiota and CRC GWAS summary statistics that were publicly available from previous studies or the corresponding authors. Specifically, GWAS summary statistics of the twinsUK study and the PopGen study can be downloaded from their respective studies in the supplementary tables (https://www.cell.com/cms/ and https://static-content.springer.com/esm/art%3A10.1038%2Fng.3695/MediaObjects/41588_2016_BFng3695_MOESM97_ESM.xlsx).

## Abbreviations

CRC: colorectal cancer
SCFA: short-chain fatty acid
MR: Mendelian randomization
IV: instrumental variable
RCT: randomized controlled trial
SNP: single nucleotide polymorphism
GWAS: genome-wide association study
UKB: UK Biobank
IRB: institutional review board
OTU: operational taxonomic unit
MAF: minor allele frequency
ICD9: International Classification of Disease diagnosis code 9
MAC: minor allele count
HRC: Haplotype Reference Consortium
SAIGE: Scalable and Accurate Implementation of GEneralized mixed model
LD: linkage disequilibrium
IVW: inverse-variance weighted
HCA: heterocyclic amine
